# Efficacy of three modern anti-diabetic drugs on survival outcomes of lung cancer patients with type 2 diabetes in China

**DOI:** 10.3389/fonc.2025.1498927

**Published:** 2025-02-18

**Authors:** Zijia Chen, Ziyi Sun, Zhongtao Zhang, Chao Lei, Zhewen Ren, Yupeng Di, Zhifei Wang

**Affiliations:** ^1^ Institute of Basic Research in Clinical Medicine, China Academy of Chinese Medical Sciences, Beijing, China; ^2^ Department of Cardiology, Guang’anmen Hospital, China Academy of Chinese Medical Sciences, Beijing, China; ^3^ Radiography Department, Hengshui Traditional Chinese Medicine Hospital, Hengshui, Hebei, China; ^4^ Division of Endocrinology and Metabolic Diseases, Department of Internal Medicine, Maastricht University Medical Center, Maastricht, Netherlands; ^5^ Department of Radiation Oncology, Air Force Medical Center, Chinese People’s Liberation Army (PLA), Beijing, China

**Keywords:** lung cancer, type 2 diabetes, real world study, anti-diabetic drugs, survival

## Abstract

**Background:**

Some anti-diabetic drugs have been proved to be a tumor suppressor or activator. The associations of three relatively new classes of anti-diabetic medications–glucagon-like peptide-1 receptor agonists (GLP-1RA), dipeptidyl peptidase 4 inhibitors (DPP-4I), and sodium-glucose cotransporter 2 inhibitors (SGLT-2I) with lung cancer prognosis remain unclear.

**Methods:**

The electronic medical data from the National Healthcare Big Data (East) Center was retrospectively analyzed. We included 11,357 newly diagnosed lung cancer patient with type 2 diabetes (T2D) between January 1st, 2020 and July 1^st^, 2023. Patients were categorized into eight groups according to the mono-or-combination therapy of GLP-1RA, DPP-4I and SGLT-2I. Disease progression and mortality risk were evaluated by cox proportional hazards analysis. Progression-free survival (PFS) and overall survival (OS) were assessed by the Kaplan-Meier (log-rank) method.

**Results:**

Lung cancer patients with T2D who were treated with SGLT-2I & GLP-1RA exhibited the lowest progression (hazard ratio [HR]: 0.37; 95% confidence interval [CI]: 0.18, 0.78) and mortality risks (HR: 0.34; 95% CI: 0.14, 0.82) as well as prolonged median PFS (1.55 years) and OS (1.62 years) among all groups. In contrast, DPP-4I monotherapy did not show benefit for progression (HR: 1.09; 95% CI: 0.98, 1.22. Median PFS: 1.41 years) and mortality risks (HR: 0.96, 95%CI: 0.84, 1.09. Median OS: 1.48 years). However, when DPP-4I was used in combination with SGLT-2I or GLP-1RA, it caused reductions in both progression and mortality risks.

**Conclusion:**

SGLT-2I & GLP-1RA dual therapy is associated with improved prognosis for lung cancer patients with concurrent T2D. DPP-4I transits from a tumor activator to suppressor when combined with other anti-diabetic drugs. Future studies are needed to examine the underlying biological mechanisms.

## Introduction

Type 2 diabetes (T2D) and cancers are both prevalent diseases worldwide. Epidemiological evidence has suggested that T2D is associated with increased incidences of multiple cancers, including lung cancer (1–5). Lung cancer is a major public health problem globally (6). In contrast to the decreasing trend in most western countries (7, 8), the incidence and mortality of lung cancer are still increasing in China. In 2022, lung cancer had the highest incidence and mortality rates among all cancers, with approximately 1,060,600 new cases and 733,300 lung cancer-related deaths reported in this country (9). These alarming numbers emphasize the need of more effective pharmacological therapies in lung cancer treatment.

With the increasing incidence T2D and obesity, these metabolic disorders are associated with chronic inflammation, insulin resistance, cell proliferation, and immune dysregulation, which have been implicated in the increased risk of both small cell lung cancer and non-small cell lung cancer (10, 11). The 5-year survival rate for lung cancer patients with T2D is only 13.1% (12). Therefore, it is meaningful to investigate the impact of pharmacotherapy on survival prognosis in lung cancer patients with T2D.

Studies have demonstrated that anti-diabetic drugs may positively influence both the risk and prognosis of lung cancer (13–16). However, most of the studies primarily focus on the effectiveness of metformin and thiazolidinedione. Until now, only a few studies have investigated the efficacy of relatively newer classes of antidiabetic medications, such as glucagon-like peptide-1 receptor agonists (GLP-1RA), dipeptidyl peptidase 4 inhibitors (DPP-4I), and sodium-glucose cotransporter 2 inhibitors (SGLT-2I) on lung cancer prognosis and these studies have all been conducted in Western countries (17–19). No research has yet examined the anti-cancer effects of these three drugs in China.

Therefore, in this study, we would like to investigate the effects of monotherapy and combination therapy with three newer anti-diabetic drugs, i.e., GLP-1RA, DPP-4I and SGLT-2I on survival outcomes of lung cancer patients with T2D in China.

## Materials and methods

### Study population

A retrospective analysis was conducted on electronic medical record data from 459,269 newly diagnosed lung cancer cases registered in the National Healthcare Big Data (East) Center form January 1^st^, 2020 to July 1^st^, 2023. The study was approved by the ethic committee of Hengshui Hospital of Chinese Medicine. Lung cancer was identified using the International Classification of Diseases 10th revision (ICD-10) codes (**Supplementary Table 1**. National RC020-ICD-10 version). Among these patients, 47,812 were also diagnosed with T2D based on ICD-10 codes (**Supplementary Table 2**). From this group, 12,648 individuals who had records of using any of the three anti-diabetic drugs (i.e., GLP-1RA, DPP-4I and SGLT-2I) were selected for further analysis. After excluding patients with survival or progression-free survival < 60 days, a final study sample of 11,357 lung cancer patients with T2D was established (**Supplementary Figure 1**).

### Anti-diabetic drugs and other variables

Patients who used any of the three anti-diabetic drugs for at least 28 continuous days were defined as users of that drug according to previous randomized controlled trials (20, 21). The treatment groups were categorized as follows: 1) DPP-4I only; 2) SGLT-2I only; 3) GLP-1RA only; 4) DPP-4I & GLP-1RA combination therapy; 5) DPP-4I & SGLT-2I combination therapy; 6) SGLT-2I & GLP-1RA combination therapy; 7) DPP-4I & GLP-1RA & SGLT-2I combination therapy.

Smoking status was classified into smoker (both current or previous smoker) and non-smoker. Drinking status was categorized as drinker (both current or previous drinker) and non-drinker. HemoglobinA1c (HbA1c), fibrinogen, prothrombin time and three cancer biomarkers–cytokeratin fragment 21-1 (CYFRA21-1), cancer antigen 125 (CA-125) and carcinoembryonic antigen (CEA) were measured in venous fasting blood samples. We also retrieved the data on family history of lung cancer, presence of other chronic diseases (kidney disease, hypertension, stroke, coronary artery disease, pulmonary disease) diagnosed by ICD-10 codes, metastasis to other organs (brain, bone, liver and kidney), diabetic complications and anti-cancer treatment measures (chemotherapy, immunotherapy, radiotherapy, targeted therapy, surgery and bevacizumab anti-angiogenic therapy). Other hypoglycemic therapies included the use of insulin secretagogues, biguanides, glucuronide inhibitors or thiazolidinedione. Pathology was divided into five categories – squamous cell carcinomas, adenocarcinoma, adenosquamous carcinoma, small cell carcinoma and large cell carcinoma.

The events of interest were lung cancer progression and all-cause mortality. The survival outcomes were measured by two commonly used indexes: progression-free survival (PFS), defined as the time from the start of treatment to the occurrence of disease progression or death (22), and overall survival (OS), defined as the time from the treatment to death (23).

### Statistical analysis

The baseline demographic characteristics, clinical characteristics and the treatment measures were presented as number (%) or as median (interquartile range) as appropriate. Comparisons between different groups were made by Kruskal-Wallis H test and chisq quare test.

The associations of different anti-diabetic regimens with disease progression and all-cause mortality were examined by cox regression analyses. The following regression models were used for the analyses: model 1: crude model; model 2: with adjustment for age and sex; model 3: with additional adjustment for smoking and drinking status; model 4: with additional adjustment for HbA1c (binary, <6.5 and ≥6.5%), kidney disease, stroke, pulmonary disease, diabetic complications, prothrombin time (<10, 10-13 and >13s), CYFRA21-1 (binary, ≤3.15 and >3.15ng/mL), CA-125 (binary, ≤35 and >35 U/mL), CEA (binary; for non-smokers: <2.5 and ≥2.5 μg/L; for smokers: < 5 and ≥ 5 μg/L), chemotherapy, immunotherapy, targeted therapy, biguanides, surgical treatment, type of pathology, and metastasis (binary, yes/no). Confounders were identified based on factors suggested to be potentially associated with the prognosis of T2D or lung cancer in previous studies (24–28), demographic characteristics, and lifestyle factors. Subsequently, the final model was determined based on the Bayesian Information Criterion. The Kaplan–Meier method was used to evaluate PFS and OS, and the log-rank test was used to compare the median survival. To make our results more robust, we defined the users as patients who used the drug for at least 14 continuous days and repeated the above analyses as sensitivity analyses.

All statistical analyses were performed with SAS (version 9.4, SAS Institute Inc. Cary, NC) and R (version 4.4.1, https://www.r-project.org/). A two-sided P value of <0.05 was considered statistically significant in all analyses.

## Results

### General characteristics of the study population

Baseline characteristics of the lung cancer patients with T2D (n = 11,357) and stratification for different treatment groups are shown in **Table 1** and **Supplementary Figure 2**. Among these patients, 5,027 did not receive any treatment. The remaining patients were treated as follows: 1,846 received monotherapy with DPP-4I, 3,070 with SGLT-2I, and 180 with GLP-1RA, 48 patients were treated with the combination therapy of DPP-4I & GLP-1RA, 912 with DPP-4I & SGLT-2I, 184 with SGLT-2I & GLP-1RA and 90 with all three drugs. The median age of the patients was 68 (interquartile range: 60,74) years old, more than 60% were males, 24.68% were smokers and 12.53% were drinkers. The median HbA1c level was 7.84% (interquartile range: 6.76%, 9.30%), and the patients had normal hemostatic functions in general. Compared with untreated patients, patients treated with anti-diabetic drugs had lower levels of cancer biomarkers. Patients treated with DPP-4I monotherapy were more likely to receive other anti-cancer treatments. More than 60% patients were adenocarcinoma in pathology and only a few of them had metastasis to other organs. Statistically significant differences were found in most characteristics among different groups except the family history of lung cancer, and brain, liver and renal metastasis. In sensitivity analyses, the number of patients receiving different therapies and their characteristics were comparable to the main analysis (**Supplementary Table 3**).

**Table 1:** Baseline characteristics of patients with lung cancer and T2D by different anti-diabetic regimens.

Characteristics	Total (n = 11357)	Without (n = 5027)	DPP-4I (n = 1846)	SGLT-2I (n = 3070)	GLP-1RA (n = 180)	DPP-4I& GLP-1RA (n = 48)	DPP-4I & SGLT-2I (n = 912)	SGLT-2I& GLP-1RA (n = 184)	DPP-4I & SGLT-2I & GLP-1RA (n = 90)	P-value
Age, years	68 (60, 74)	68 (61, 74)	68 (61, 74)	67 (60, 73)	60 (47.7, 68)	57.5 (42, 73)	67 (60, 73)	59 (50, 67.2)	62.5 (56, 68)	<0.001
Males	7078 (62.32)	3044 (60.55)	1135 (61.48)	1999 (65.11)	101 (56.11)	19 (39.58)	606 (66.45)	114 (61.96)	60 (66.67)	<0.001
Smoker	2803 (24.68)	1203 (23.93)	473 (25.62)	754 (24.56)	34 (18.89)	10 (20.83)	272 (29.82)	33 (17.93)	24 (26.67)	0.001
Drinker	1423 (12.53)	604 (12.02)	219 (11.86)	398 (12.96)	21 (11.67)	4 (8.33)	140 (15.35)	18 (9.78)	19 (21.11)	0.015
Family history of lung cancer	152 (1.34)	65 (1.29)	29 (1.57)	39 (1.27)	1 (0.56)	0 (0.00)	13 (1.43)	3 (1.63)	2 (2.22)	0.864
Kidney disease	739 (6.51)	293 (5.83)	153 (8.29)	183 (5.96)	17 (9.44)	4 (8.33)	66 (7.24)	9 (4.89)	14 (15.56)	<0.001
Hypertension	7118 (62.68)	3036 (60.39)	1130 (61.21)	2031 (66.16)	105 (58.33)	24 (50.00)	602 (66.01)	128 (69.57)	62 (68.89)	<0.001
Coronary heart disease	2688 (23.67)	1003 (19.95)	330 (17.88)	982 (31.99)	32 (17.78)	6 (12.50)	255 (27.96)	48 (26.09)	32 (35.56)	<0.001
Stroke	3298 (29.04)	1384 (27.53)	492 (26.65)	1019 (33.19)	36 (20.00)	13 (27.08)	265 (29.06)	58 (31.52)	31 (34.44)	<0.001
Pulmonary disease	791 (6.96)	330 (6.56)	162 (8.78)	217 (7.07)	6 (3.33)	2 (4.17)	66 (7.24)	4 (2.17)	4 (4.44)	0.002
Diabetic complications	1329 (11.70)	464 (9.23)	203 (11.00)	365 (11.89)	37 (20.56)	14 (29.17)	157 (17.21)	59 (32.07)	30 (33.33)	<0.001
HbA1c, %	7.84 (6.76, 9.30)	7.90 (6.80, 9.34)	7.50 (6.50, 8.90)	7.90 (6.80, 9.30)	8.39 (7.00, 10.00)	8.40 (7.21, 10.50)	7.80 (6.80, 9.13)	8.35 (7.00, 9.75)	8.15 (7.24, 9.40)	<0.001
Fibrinogen, g/L	3.36 (2.69, 4.38)	3.42 (2.73, 4.44)	3.35 (2.67, 4.40)	3.30 (2.66, 4.29)	3.24 (2.61, 4.20)	3.40 (2.73, 4.36)	3.29 (2.65, 4.25)	3.30 (2.81, 4.08)	3.09 (2.50, 3.81)	<0.001
Prothrombin time, s	11.60 (10.90, 12.86)	11.70 (10.90, 12.90)	11.70 (10.90, 12.90)	11.60 (10.90, 12.80)	11.50 (10.80, 13.31)	11.58 (10.96, 12.80)	11.50 (10.86, 12.60)	11.50 (10.80, 12.49)	11.35 (10.70, 12.30)	0.019
CYFRA21-1, ng/mL	5.15 (2.59, 11.94)	5.51 (2.67, 12.50)	5.00 (2.65, 11.83)	4.86 (2.52, 11.14)	6.60 (2.76, 12.88)	5.07 (2.24, 9.39)	4.29 (2.31, 10.57)	5.27 (2.32, 11.56)	4.24 (2.11, 11.18)	<0.001
CA-125, U/mL	90.70 (19.03, 188.49)	92.71 (19.81, 191.44)	92.57 (20.25, 189.56)	86.77 (16.98, 182.13)	116.72 (40.18, 192.33)	56.98 (27.19, 167.18)	82.30 (17.52, 190.04)	98.19 (20.50, 192.70)	86.73 (14.21, 157.83)	0.035
CEA, μg/L	3.83 (2.11, 22.90)	4.31 (2.25, 38.52)	3.80 (2.10, 22.58)	3.58 (2.05, 16.94)	3.10 (1.89, 6.24)	2.94 (1.75, 5.32)	3.30 (1.89, 8.46)	2.71 (1.77, 4.13)	2.59 (1.85, 4.76)	<0.001
Chemotherapy	4169 (36.71)	1697 (33.76)	823 (44.58)	1169 (38.08)	26 (14.44)	11 (22.92)	387 (42.43)	33 (17.93)	23 (25.56)	<0.001
Radiotherapy	1302 (11.46)	549 (10.92)	262 (14.19)	351 (11.43)	6 (3.33)	4 (8.33)	113 (12.39)	10 (5.43)	7 (7.78)	<0.001
Immunotherapy	981 (8.64)	404 (8.04)	221 (11.97)	254 (8.27)	5 (2.78)	2 (4.17)	84 (9.21)	8 (4.35)	3 (3.33)	<0.001
Targeted therapy	1242 (10.94)	533 (10.60)	269 (14.57)	316 (10.29)	3 (1.67)	6 (12.50)	108 (11.84)	3 (1.63)	4 (4.44)	<0.001
Bevacizumab anti-angiogenic therapy	534 (4.70)	228 (4.54)	122 (6.61)	137 (4.46)	1 (0.56)	2 (4.17)	41 (4.50)	2 (1.09)	1 (1.11)	<0.001
Insulin secretagogues	3899 (34.33)	1591 (31.65)	623 (33.75)	1161 (37.82)	34 (18.89)	16 (33.33)	379 (41.56)	55 (29.89)	40 (44.44)	<0.001
Biguanides	7376 (64.95)	3026 (60.19)	1134 (61.43)	2200 (71.66)	124 (68.89)	34 (70.83)	625 (68.53)	156 (84.78)	77 (85.56)	<0.001
Glucuronide inhibitors	4605 (40.55)	1868 (37.16)	790 (42.80)	1325 (43.16)	49 (27.22)	22 (45.83)	412 (45.18)	83 (45.11)	56 (62.22)	<0.001
Thiazolidinedione	532 (4.68)	182 (3.62)	82 (4.44)	166 (5.41)	5 (2.78)	3 (6.25)	70 (7.68)	13 (7.07)	11 (12.22)	<0.001
Surgical treatment	3295 (29.01)	1382 (27.49)	582 (31.53)	896 (29.19)	37 (20.56)	14 (29.17)	313 (34.32)	44 (23.91)	27 (30.00)	<0.001
Type of pathology										<0.001
Squamous cell carcinomas	2552 (22.47)	1132 (22.52)	430 (23.29)	694 (22.61)	27 (15.00)	2 (4.17)	211 (23.14)	35 (19.02)	21 (23.33)	
Adenocarcinoma	7716 (67.94)	3400 (67.63)	1224 (66.31)	2101 (68.44)	143 (79.44)	46 (95.83)	596 (65.35)	143 (77.72)	63 (70.00)	
Adenosquamous carcinoma	96 (0.85)	42 (0.84)	22 (1.19)	29 (0.94)	1 (0.56)	0 (0.00)	2 (0.22)	0 (0.00)	0 (0.00)	
Small cell carcinoma	959 (8.44)	437 (8.69)	165 (8.94)	235 (7.65)	9 (5.00)	0 (0.00)	101 (11.07)	6 (3.26)	6 (6.67)	
Large cell carcinoma	34 (0.30)	16 (0.32)	5 (0.27)	11 (0.36)	0 (0.00)	0 (0.00)	2 (0.22)	0 (0.00)	0 (0.00)	
Brain metastasis	141 (1.24)	74 (1.47)	21 (1.14)	34 (1.11)	1 (0.56)	2 (4.17)	8 (0.88)	0 (0.00)	1 (1.11)	0.170
Bone metastasis	407 (3.58)	181 (3.60)	77 (4.17)	112 (3.65)	0 (0.00)	1 (2.08)	34 (3.73)	1 (0.54)	1 (1.11)	0.031
Liver metastasis	151 (1.33)	69 (1.37)	24 (1.30)	34 (1.11)	1 (0.56)	1 (2.08)	20 (2.19)	2 (1.09)	0 (0.00)	0.300
Renal metastasis	72 (0.63)	46 (0.92)	8 (0.43)	12 (0.39)	1 (0.56)	0 (0.00)	5 (0.55)	0 (0.00)	0 (0.00)	0.134

Data are reported as median (interquartile range) or number (%) as appropriate. Comparisons between different groups were made by Kruskal-Wallis H test or chisq square test.

CA-125, Carbohydrate antigen 125; CEA, Carcinoembryonic antigen; CYFRA21-1, Cytokeratin fragment 21-1; HbA1c, Hemoglobin A1c.

### Association of anti-diabetic regimens with disease progression

**Figure 1:**
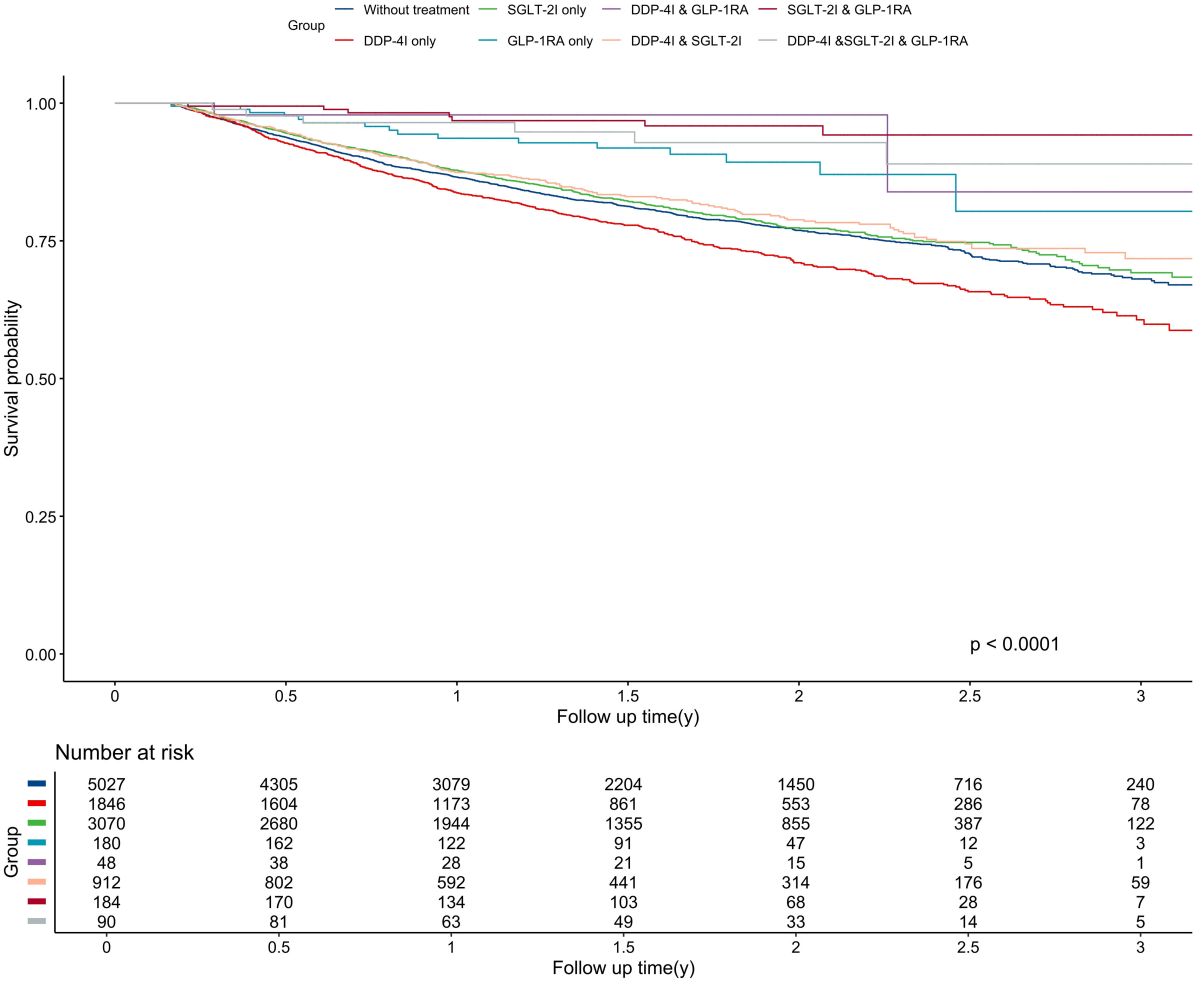
Kaplan-Meier curve for progression-free survival among different treatment groups. The unit of follow-up time was year. DPP-4I, Dipeptidyl peptidase 4 inhibitors; GLP-1RA, Glucagon-like peptide-1 receptor agonists; SGLT-2I, Sodium-glucose cotransporter 2 inhibitors.

The median follow-up was 1.37 years, generating 16,649 person-years of follow-up. During this time, 2,167 patients had disease progression, resulting in an overall disease progression rate of 13.02 per 100 person-years. Patients receiving DPP-4I monotherapy had the highest disease progression rate (16.62 per 100 person-years), which was even higher than that of the untreated group (13.32 per 100 person-years). In contrast, patients treated with SGLT-2I & GLP-1RA combination therapy had the lowest disease progression rate at 2.33 per 100 person-years. There were significant differences in PFS among different anti-diabetic regimens (P<0.001) (**Figure 1**). The longest and shortest PFS were observed in the SGLT-2I & GLP-1RA combination therapy group (1.55 years) and DPP-4I monotherapy group (1.41 years) respectively.

**Figure 2:**
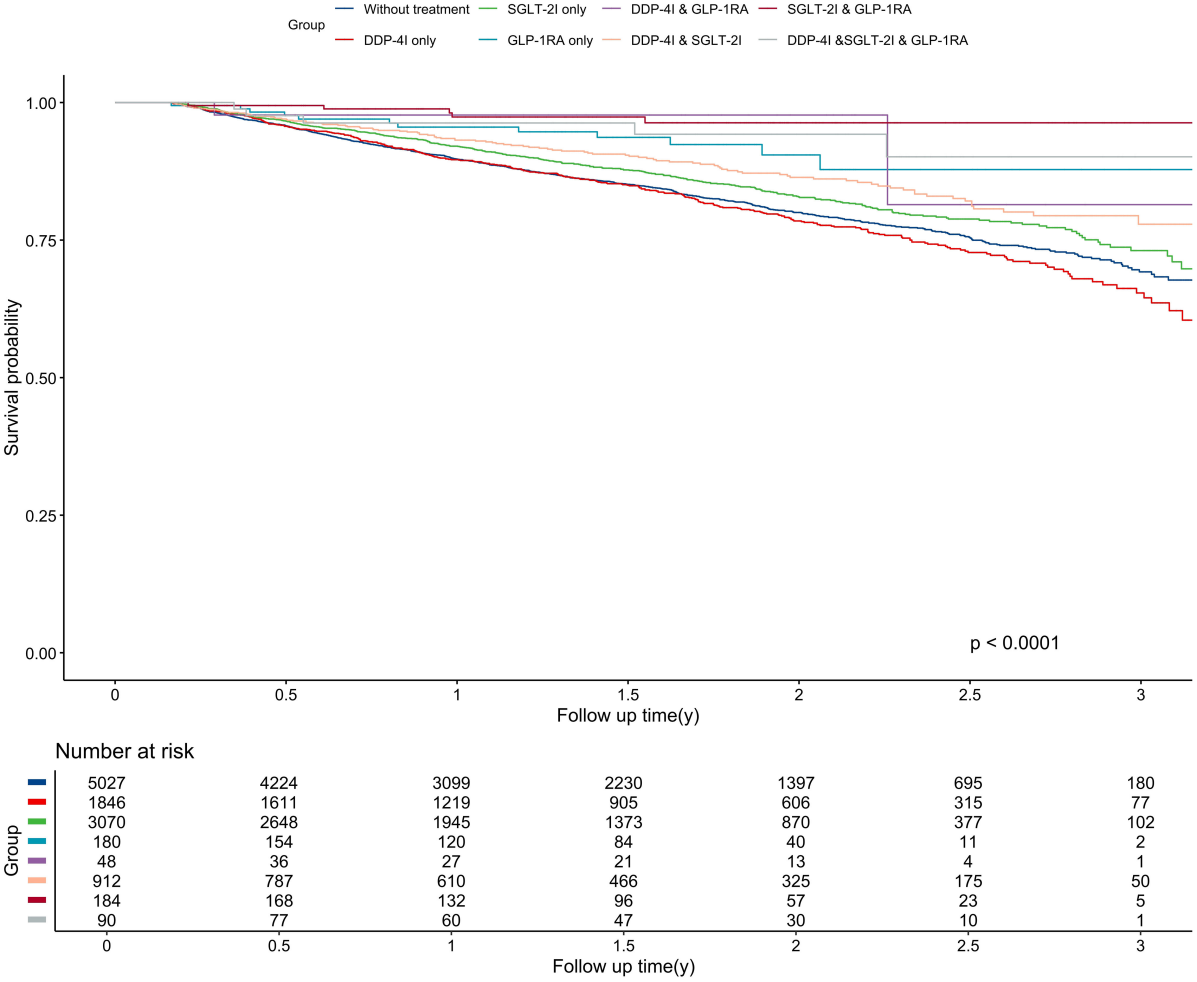
Kaplan-Meier curve for overall survival among different treatment groups. The unit of follow-up time was year. DPP-4I, Dipeptidyl peptidase 4 inhibitors; GLP-1RA, Glucagon-like peptide-1 receptor agonists; SGLT-2I, Sodium-glucose cotransporter 2 inhibitors.

**Figure 2** showed that those treated with SGLT-2I & GLP-1RA combination therapy had the lowest risk of disease progression, with a 63% decrease in risk compared to untreated patients (hazard ratio [HR]: 0.37; 95% confidence interval [CI]: 0.18, 0.78 in the fully adjusted model). However, patients receiving the DPP-4I monotherapy had a 9% higher disease progression risk (HR: 1.09; 95% CI: 0.98, 1.22) than the untreated patients although the result was not statistically significant after full adjustment. Notably, this was also the only treatment group with a higher disease progression risk than the untreated group. Of interest, when DPP-4I was used in combination with GLP-1RA or SLGT-2I, the disease progression risk decreased (HR: 0.52; 95% CI: 0.17, 1.63 for DPP-4I & GLP-1RA combination therapy; HR: 0.93; 95% CI: 0.79, 1.10 for DPP-4I & SGLT-2I combination therapy). Moreover, patients receiving the combination of the DPP-4I, SGLT-2I and GLP-1RA were observed to have a reduced progression risk in both the univariate model and the model adjusted for age, gender, and lifestyle. However, the association became non-significant after further adjustment for biochemical indicators. Sensitivity analyses yielded similar results (**Supplementary Table 4**, **Supplementary Figure 3**).

### Association of anti-diabetic regimens with death

**Figure 3:**
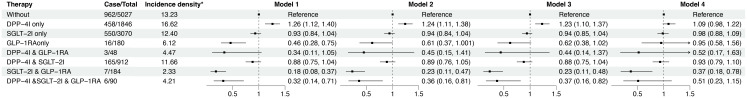
The progression risk among different anti-diabetic treatment groups. * The unit of incidence density is per 100 person years. Model 1 was crude model; Model 2 adjusted for age and sex; Model 3 further adjusted for smoking and drinking status; Model 4 further adjusted for HbA1c (<6.5, ≥ 6.5%), kidney disease, stroke, pulmonary disease, diabetic complications, prothrombin time (<10, 10-13, >13s), CYFRA21-1 (≤3.15, >3.15ng/mL), CA-125 (≤35, >35 U/mL), CEA (for non-smokers: <2.5, ≥2.5 μg/L; for smokers: < 5, ≥ 5 μg/L), chemotherapy, immunotherapy, targeted therapy, biguanides, surgical treatment, type of pathology, and metastasis (with/without). CA-125, Carbohydrate antigen 125; CEA, Carcinoembryonic antigen; CYFRA21-1, Cytokeratin fragment 21-1; HbA1c, Hemoglobin A1c.

The median follow-up was 1.39 years, generating 16,647 person-years of follow-up. During the study period, 1,676 patients died either from lung cancer-related events or other causes, resulting in a mortality rate of 10.07 per 100 person-years. Patients who were treated with DPP-4I monotherapy still had the highest mortality risk, with a rate of 12.04 per 100 person-years, which was still higher than the mortality of untreated patients (11.04 per 100 person-years). Conversely, The SGLT-2I & GLP-1RA combination therapy group had the lowest mortality rate at 1.75 per 100 person-years. Significant differences were shown in OS among different anti-diabetic regimens (P<0.001) (**Figure 3**). Similarly, The SGLT-2I & GLP-1RA combination therapy group had the longest OS with 1.62 years, whereas the DPP-4I group had the shortest OS with 1.48 years.

**Figure 4:**
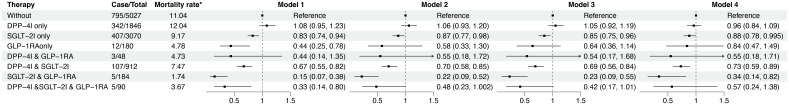
The mortality risk among different anti-diabetic treatment groups. * The unit of mortality rate is per 100 person years. Model 1 was crude model; Model 2 adjusted for age and sex; Model 3 further adjusted for smoking and drinking status; Model 4 further adjusted for HbA1c (<6.5, ≥ 6.5%), kidney disease, stroke, pulmonary disease, diabetic complications, prothrombin time (<10, 10-13, >13s), CYFRA21-1 (≤3.15, >3.15ng/mL), CA-125 (≤35, >35 U/mL), CEA (for non-smokers: <2.5, ≥2.5 μg/L; for smokers: < 5, ≥ 5 μg/L), chemotherapy, immunotherapy, targeted therapy, biguanides, surgical treatment, type of pathology, and metastasis (with/without). CA-125, Carbohydrate antigen 125; CEA, Carcinoembryonic antigen; CYFRA21-1, Cytokeratin fragment 21-1; HbA1c, Hemoglobin A1c.

The results showed that patients receiving SGLT-2I & GLP-1RA combination therapy had a 66% lower disease progression rate (HR: 0.34; 95% CI: 0.14, 0.82) than the untreated group. DPP-4I monotherapy did not demonstrate statistically significant advantage for mortality (HR: 0.96, 95%CI: 0.84, 1.09), whereas when DPP-4I was used in combination with GLP-1RA/SLGT-2I, the mortality risk decreased (HR: 0.55; 95% CI: 0.18, 1.71 for DPP-4I & GLP-1RA combination therapy; HR: 0.73; 95% CI: 0.59, 0.89 for DPP-4I & SGLT-2I combination therapy). Moreover, patients receiving the combination of the DPP-4I, SGLT-2I and GLP-1RA were observed to have a reduced mortality risk in both the univariate model. However, the association became non-significant after further adjustment for age and gender (**Figure 4**). The reclassification of the days for medication use did not materially change the result in sensitivity analyses (**Supplementary Table 5**, **Supplementary Figure 4**).

## Discussion

In this study, we found that the combination therapy of SGLT-2I and GLP-1RA substantially reduced the disease progression and mortality risks and prolonged the PFS and OS in lung cancer patients with T2D. In contrast, DPP-4I, when used alone, showed a negative trend for the prognosis of lung cancer. However, when DPP-4I was used in combination with either SGLT-2I or GLP-1RA, it showed clinical benefit.

To the best of our knowledge, this is the first study that reports the effect of SGLT-2I & GLP-1RA combination therapy on the survival outcomes of lung cancer. A previous study suggested that SGLT-2I is associated with a decreased mortality risk of non-small cell lung cancer (18). Our study aligns with and further extends this finding. First, we were able to include disease progression as another survival outcome, which showed similar results. Second, our results not only showed the survival advantage of SGLT-2I on lung cancer, but also indicated that the combination therapy of SGLT-2I&GLP-1RA may confer a greater survival benefit compared to SGLT-2I monotherapy. These results might be explained by the complementary mechanisms of these two drugs. SGLT-2I enhance urinary glucose excretion (29, 30), thereby reducing glucose availability to lung cancer cells. This decreased glucose uptake and utilization impairs the growth and survival of cancer cells, potentially slowing tumor progression (31, 32). Meanwhile, GLP-1RA may have synergistic effect by reduces proinflammatory markers (33–35), which could potentially reduce systemic inflammation associated with cancer progression. This promising finding warrants further research to investigate the safety and adverse events of this combination regimen in cancer treatment.

The role of DPP-4I in the natural history of lung cancer biology is still on debate (36). An *in vitro* study done by Jang et al. showed that treatment with DPP-4I vildagliptin significantly suppresses lung cancer growth by activating macrophages and NK cells (37), but Wang H and colleagues found that DPP-4I saxagliptin and sitagliptin increase cell migration and invasion of multiple cancer cell lines including lung A549 (38). These contradictory findings are further complicated by epidemiological and clinical studies. Previous investigations have reported improved OS and PFS in lung cancer patients treated with DPP-4I (17, 19). However, our findings differ from these earlier observations. The discrepancies between our results and previously reported outcomes may be attributed to several factors that may influence the efficacy of DPP-4I, such as variations in patient populations, differences in treatment durations, or the specific types of DPP-4Is evaluated. These conflicting findings highlight the necessity for further research to better understand the factors that affect the efficacy of DPP-4I in lung cancer progression and treatment. Nonetheless, one of our results is consistent with the previous finding (17) – the combination of DPP-4I with other antidiabetic drugs (e.g., metformin, GLP-1RA and SGLT-2I) offers greater survival benefits than DPP-4I monotherapy. The consistency underscores the potential significance of combination therapy strategies in enhancing the therapeutic efficacy of DPP-4I in lung cancer. Thus far, the underlying mechanisms for this intriguing finding remain unclear, highlighting the need for further experimental studies to uncover them.

Although there is controversy regarding the association between DPP-4I and the progression of lung cancer (39), previous observational studies have shown that among diabetic patients, compared with the SGLT-2I treatment regimen, DPP-4I treatment increases the risk of new-onset tumors (40). In addition, basic research also provides evidence to support the findings of this study. The research on breast cancer by Kawakita et al. has shown that DPP-4I promotes epithelial-mesenchymal transition through the CXCL12/CXCR4/mTOR axis, thereby inducing cancer progression and metastasis (41). The research on lung cancer by Wesley et al. pointed out that DPP-4 has tumor suppressor activity, and its inhibition may lead to uncontrolled growth of lung tissue (42). The results of the above studies all support the promoting effect of DPP-4I alone on the poor prognosis of lung cancer found in this study.

When DPP - 4I is combined with GLP - 1RA or SGLT - 2I, GLP - 1RA or SGLT - 2I activates the AMPK pathway (43, 44), thereby blocking the mTOR pathway, and subsequently blocking the DPP - 4I - induced epithelial - mesenchymal transition. This may be the potential mechanism underlying the improved prognosis of lung cancer patients with T2D observed in this study when used in combination with SGLT-2I or GLP-1RA.

This study has the strength of using the large-scale data from electronic medical record database, which may reduce the potential of selection bias. The comprehensive pharmacological data allows us to investigate the association between anti-diabetic drugs and the prognosis of lung cancer. However, several limitations of this study should also be considered. This study is retrospective in nature, inherently carrying the typical limitations of a retrospective study design. One of the major drawbacks is the potential for confounding factors. For instance, due to the limitations of data availability, we did not include the body mass index, an indicator closely related to medication and the prognosis of lung cancer patients. Past evidence indicates that the hypoglycemic regimen of GLP - 1RA may cause weight loss (45), and weight loss is associated with an increased risk of death for patients with lung cancer (46). This suggests that we may have underestimated the therapeutic effect of GLP - 1RA, and more studies are needed for confirmation in the future. Also, this study design prevents us from drawing direct causal inferences regarding the use of anti-diabetic drugs and survival outcomes. Moreover, the sample size is another limitation. Although the total sample size is more than ten thousand, the number of patients using specific regimen, e.g., DPP-4I & GLP-1RA is relatively low (n = 48) which may affect the accuracy of the results. Despite this, our results remained robust in sensitivity analyses.

In conclusion, the present study demonstrates that SGLT-2I & GLP-1RA combination has great potential in lung cancer treatment. DPP-4I monotherapy shows a trend for pro-cancer effect, however, it becomes more effective when used in combination with SGLT-2I or GLP-1RA.

## Data Availability

The raw data supporting the conclusions of this article will be made available by the authors, without undue reservation.
