# Supplementary material for: Efficacy of three modern anti-diabetic drugs on survival outcomes of lung cancer patients with type 2 diabetes in China
Source: Front Oncol. 2025 Feb 18;15:1498927. doi: 10.3389/fonc.2025.1498927 (PMC11876025; doi:10.3389/fonc.2025.1498927)
Supplement: Supplementary file 1 [file DataSheet1.docx]

**Supplementary Figure**

**
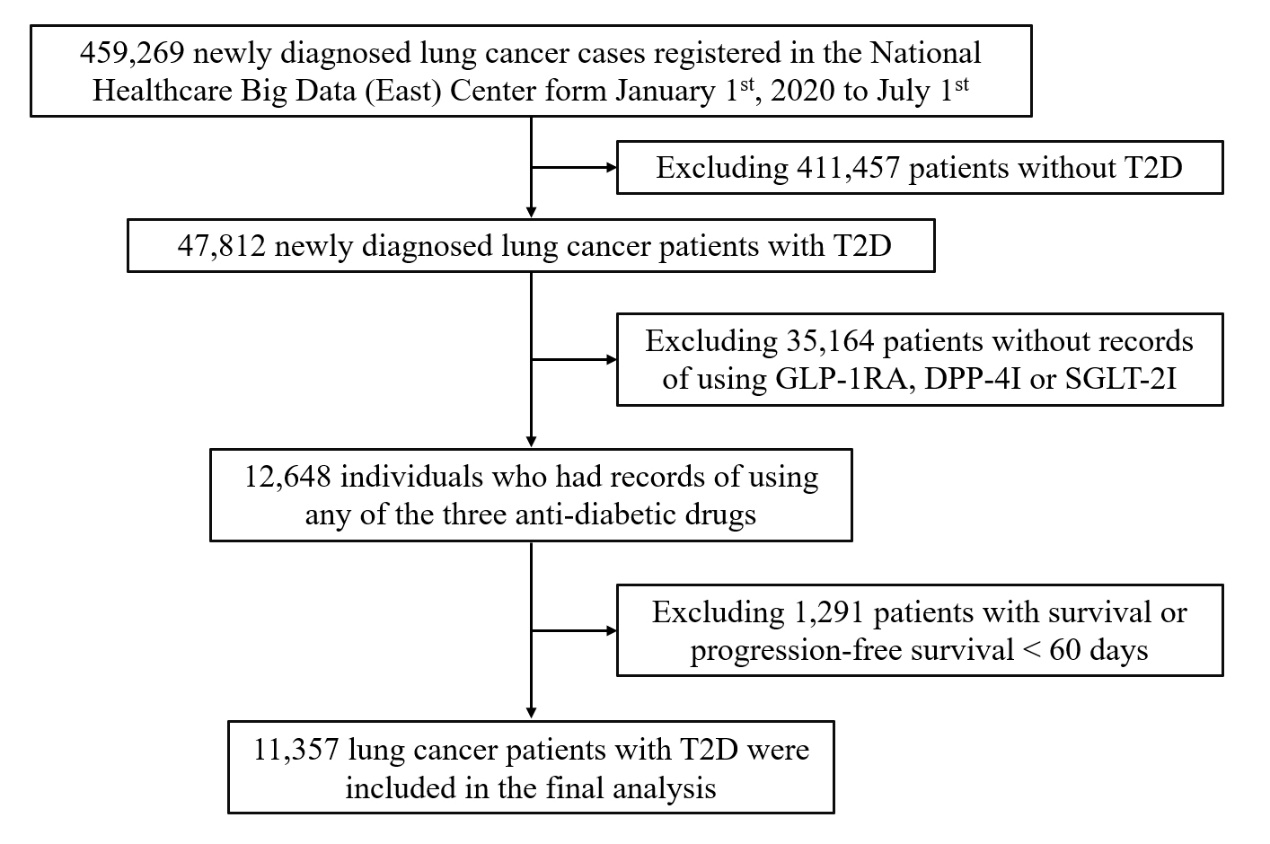
**

Supplementary Figure 1 Flowchart for the recruitment of the patients.

Abbreviations: DPP-4I, Dipeptidyl peptidase 4 inhibitors; GLP-1RA, Glucagon-like peptide-1 receptor agonists; SGLT-2I , Sodium-glucose cotransporter 2 inhibitors; T2D, Type 2 diabetes.


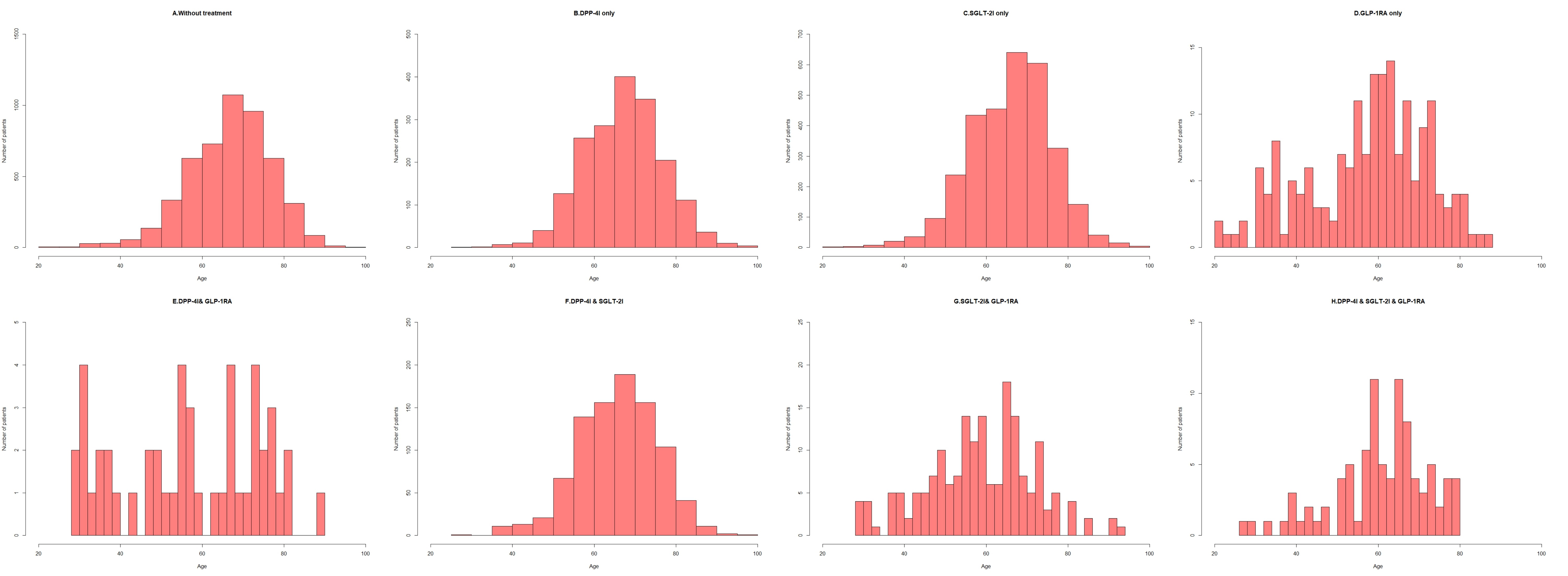


Supplementary Figure 2 Distribution of age across different treatment groups





Supplementary Figure 3 Kaplan-Meier curve for progression-free survival among different treatment groups (treatment defined as at least 14 days of continuous use).

The unit of follow-up time was year.

Abbreviations: DDP-4I, Dipeptidyl peptidase 4 inhibitors; GLP-1RA, Glucagon-like peptide-1 receptor agonists; SGLT-2I, Sodium-glucose cotransporter 2 inhibitors.





Supplementary Figure 4 Kaplan-Meier curve for overall survival among different treatment groups (treatment defined as at least 14 days of continuous use).

The unit of follow-up time was year.

Abbreviations: DDP-4I, Dipeptidyl peptidase 4 inhibitors; GLP-1RA, Glucagon-like peptide-1 receptor agonists; SGLT-2I, Sodium-glucose cotransporter 2 inhibitors.
